# The anti-proliferative effects of a frankincense extract in a window of opportunity phase ia clinical trial for patients with breast cancer

**DOI:** 10.1007/s10549-023-07215-4

**Published:** 2024-01-09

**Authors:** Ingrid V. Bonilla Valente, Denise Garcia, Andrea Abbott, Laura Spruill, Julie Siegel, Jessica Forcucci, George Hanna, Rupak Mukherjee, Mark Hamann, Eleanor Hilliard, Mark Lockett, David J. Cole, Nancy Klauber-DeMore

**Affiliations:** 1 Department of Surgery, Medical University of South Carolina, Charleston, SC USA; 2 Department of Pathology, Medical University of South Carolina, Charleston, SC USA; 3 College of Pharmacy Drug Discovery and Biomedical Sciences, Medical University of South Carolina, Charleston, SC USA; 4 Medical University of South Carolina, MSC 295, Room 240, 114 Doughty Street, Charleston, SC 29425 USA

**Keywords:** *Boswellia serrata*, Breast neoplasm, Natural products, KI67

## Abstract

**Purpose:**

Boswellic acids, active components of frankincense, suppress tumor proliferation in vitro with a strong clinical trial safety profile in patients with inflammatory diseases. We performed a Phase Ia window of opportunity trial of *Boswellia serrata* (*B. serrata*) in patients with breast cancer to evaluate its biologic activity and safety.

**Methods:**

Patients with invasive breast cancer were treated pre-operatively with *B. Serrata* (2400 mg/day PO) until the night before surgery for a median of 11 days (SD 6 days; range: 5–23 days). Paraffin-embedded sections from pretreatment diagnostic core biopsies and post-treatment surgical excisions were evaluated using a tunnel assay and immunohistochemistry staining with Ki-67 antibodies. A non-intervention retrospective control arm consisting of core and surgical tissue specimens from untreated patients was used to compare patients treated with *B. Serrata*. The change in proliferation and apoptosis between diagnostic core specimens and surgical specimens was compared between the control and treatment groups using a two-tailed paired t-test.

**Results:**

Twenty-two patients were enrolled, of which 20 received treatment, and 18 had sufficient tissue for IHC. There was an increase in percent change in proliferation from core biopsy to surgical excision in the control group (*n* = 18) of 54.6 ± 21.4%. In the *B. serrata*-treated group there was a reduction in proliferation between core biopsy and excision (*n* = 18) of 13.8 ± 11.7%. This difference was statistically significant between the control and *B. serrata*-treated groups (*p* = 0.008). There was no difference in change in apoptosis. There were no serious adverse events related to the drug.

**Conclusion:**

*Boswellia serrata* inhibited breast cancer proliferation and was well-tolerated in a Phase Ia window of opportunity trial.

## Background

Despite the advances in breast cancer treatments and early detection methods that improve breast cancer prognosis, many current treatments have long-term adverse side effects, including neuropathy [1], neutropenia [2], alopecia [3], lymphedema [4], cardiovascular disease [5], and increased risk of developing other cancers [6]. Additionally, cancer recurrence in this patient population is not unusual, and so the development of effective and safe therapies is needed. A potential opportunity exists in the use of complementary and alternative therapies, such as natural plant products. The biological activity of botanical products often lacks a strong basis in scientific or clinical evidence and effective methodologies are needed to characterize their utility and application.

**Fig. 1:**
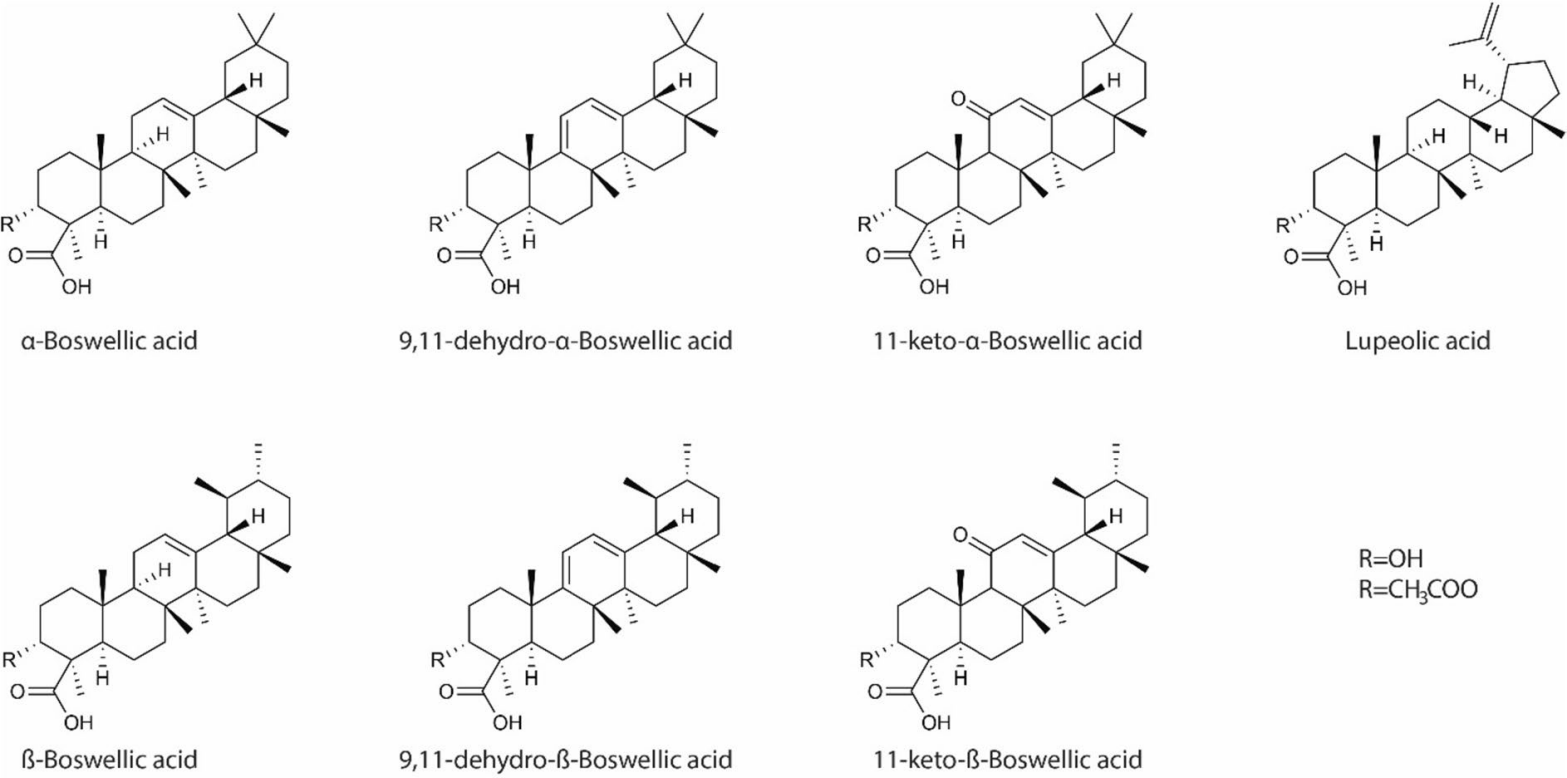
Major types of boswellic acids in *B. serrate*

Previous plant-derived highly successful single chemical entities used in medicine include digitoxin [7], aspirin[8], lovastatin[9], topotecan[10], irinotecan [10], and paclitaxel [11]. A potential botanical that warrants further scientific investigation is *Boswellia serrata* (*B. serrata*), the active component of frankincense. Frankincense is a hardened gum-like material (resin) that comes from the trunk of trees of the Boswellia genus. The gum resin of *B. serrata* contains at least 12 different types of boswellic acids (BAs), but among these the six major acids identified are α and β-boswellic acids (BA), acetylated α and β-boswellic acids (ABA), 11-keto-β-boswellic acid (KBA), and 3-O-acetyl-11-keto-β-boswellic acid (AKBA) (Fig. 1)—Boswellic acids, are reported to have properties that help suppress tumor activity and interact in various signaling pathways involved in apoptosis, cell proliferation, and angiogenesis [12, 13]. They have anti-proliferative and pro-apoptotic properties in multiple human cancer cell lines in vitro including meningioma [14], leukemia [15], melanoma, fibrosarcoma [16], and cancers of the colon [17], brain [14] breast [18], and prostate [19]. Oral AKBA inhibited human prostate tumor growth in xenograft mice through inhibition of angiogenesis induced by VEGFR2 signaling pathways [19]. Additionally, clinical trials have shown that *B. serrata* is effective in inflammatory conditions such as arthritis [20], bronchial asthma [13, 21], and gingivitis[22] with a strong safety profile.

An emerging highly significant and innovative approach to the assessment of botanicals involves the application of window of opportunity clinical trials (window trials) [23]. Window of opportunity trials study the treatment of a drug between diagnosis and the planned, standard treatment. These trials are uniquely suited to the assessment of botanicals since they can provide early insight into potential biologic activity in humans. During this time, researchers can evaluate target modulation after drug exposure and understand an agent’s biologic effect early in its development. The advantage of window trials is that they allow for rapid evaluation of novel agents in patients who already have surgery planned as their primary therapy. In this setting the agents may be tested in patients who have not been exposed to other forms of treatment [24]. Unlike the more traditional neoadjuvant trials, correlative studies are primary endpoints rather than therapeutic benefit. Several window trials have used proliferative marker Ki-67 as an appropriate primary end-point to evaluate the outcome in post-treatment breast cancer patients and as potential early determinant of long-term benefit of endocrine therapy [25–27].—expression level is the most widely used surrogate marker of effectiveness based on data that shows its association with long-term outcomes [24]. With a direct trial in humans, investigators have the opportunity to make an early determination of whether a drug is worth further investigation. Given the pre-clinical data of tumor efficacy and clinical data demonstrating a favorable safety profile, we hypothesized that *B. serrata* had in vivo efficacy in human breast cancer and so pursued a Phase Ia window of opportunity trial.

## Methods

### Study participants and eligibility

This was an open-label, single center, single arm, prospective Phase Ia window of opportunity trial. The clinical trial was registered on ClinicalTrials.gov. Identifier NCT03149081 (Date of registration May 11, 2017), and the Institutional Review Board at the Medical University of South Carolina approved this study. Informed consent was obtained from each patient. This paper reports the results of the patients with invasive breast cancer. Eligibility criteria included the following: (1) histological confirmation (by diagnostic core biopsy) of invasive breast cancer (stages I, II, or III) with primary tumor(s) ≥ 1.0 cm on mammogram, ultrasound, breast MRI, or physical exam; (2) patients scheduled for surgery no less than five days from the planned start of day one of *B.serrata* treatment and no more than 56 days from the planned start of day one; (3) age ≥ 18 years; (4) Eastern Cooperative Oncology Group status 0 or 1; (5) women of child-bearing potential must agree to use an approved form of birth control and have negative pregnancy test; (6) adequate hematologic and end organ function, defined by ANC ≥ 1.5 × 10^9^/L, platelet count ≥ 100 × 10^9^/L, hemoglobin ≥ 9 g/dL, albumin ≥ 2.5 g/dL, bilirubin ≤ 1.5 × the upper limit of normal (ULN), AST, ALT, and alkaline phosphatase ≤ 3 × ULN, serum creatinine ≤ 1.5 × ULN or creatinine clearance ≥ 40 mL/min; and (7) at least six sections of unstained slides available from the core biopsy for use for the study.

Exclusion criteria were as follows: (1) patients undergoing neoadjuvant chemotherapy or endocrine therapy; (2) subjects with end-stage kidney disease and/or grade II liver dysfunction; (3) active or history of deep vein thrombosis; (4) history of coagulopathies or hematological disorders; (5) patients already taking anti-coagulant, platelet inhibitors, 5-lipoxygenase inhibitors, drugs that interact with OATP1B3 (an anion transporter), MRP2 (a multidrug resistant protein), and/or P-glycoprotein were also ineligible for study participation as these interact with Boswellia [28, 29] [30] (Table 1 for all prohibitive medications). A non-intervention control arm was included that consisted of core and surgical tissue specimens from 20 untreated patients obtained from the Hollings Cancer Center Biorepository to compare core biopsy and surgical excision in this non treatment set of patients to the treatment arm. This group of patients had pathologic T1c-T2 invasive breast cancer, hormone receptor positive and Her2neu negative, and had not received neoadjuvant chemotherapy or endocrine therapy. No clinical data was requested, and specimens were de-identified.

**Table 1:** Prohibited medications that exclude patients from participating in the trial

5-Lipoxygenase inhibitors	Anti-coagulants or anti-platelet drugs	OATP1B3, MRP2, and/or PgP substrates
Zileuton	Coumarins and indandiones Factor Xa inhibitors Heparins Thrombin inhibitors	Amiodarone Asunaprevir Atazanavir Atovarstatin Benzylpenicillin Bosentan Carvedilol Cerivastatin Cholecystokinin octapeptide (CCK-8) Clarithomycin Cyclosporine Dabigatran Danoprevir Digoxin Docetaxel Dronedarone Elacridar Erythomycin Estradiol-17β-glucuronide Estrone-3-sulfate Fexofenadine Gemfibrozil Glyburide Itraconazole Ketoconazole Lapatinib Loperamide Lopinavir Methotrexate 2-Amino-1-methyl-6-Phenylimidazo[4,5-b]pyridine (PhIP)

The primary endpoint of this study was to determine whether oral administration of *B. serrata* causes biological changes related to cell proliferation (Ki-67) and apoptosis (DNA fragmentation) in primary tumors of breast cancer patients. The secondary endpoint was to determine the safety and toxicity of oral *B. serrata* in patients with breast cancer.

### Drug information

BosPure® 400 mg was supplied for the study by Arjuna Natural Pvt. Ltd (Aluva, India). It is an oral non-GMO extract of the *B. serrata* resin that is standardized to contain no less than 70% triterpenic boswellic acids. Per the manufacturer, each vegetarian capsule contains up to 35% boswellic acids and 40% total organic acids, and the boswellic acids that make up 35% of the drug are: acetyl-alpha boswellic acid (8–14%), acetyl-beta- boswellic acid (17–25%), and acetyl-11-keto-beta boswellic acid (AKBA, at least 10%).

In previous clinical studies the major toxic effects of *B. serrata* that limited dose were gastrointestinal [31, 32]. Patients were instructed to contact the study team if they experienced any intolerable side effect, and the dose was withheld for up to three days until the adverse event improved or resolved. If the event symptoms did not improve or resolve within three days, the patient was discontinued from study.

### Treatment administration and compliance

**Fig. 2:**
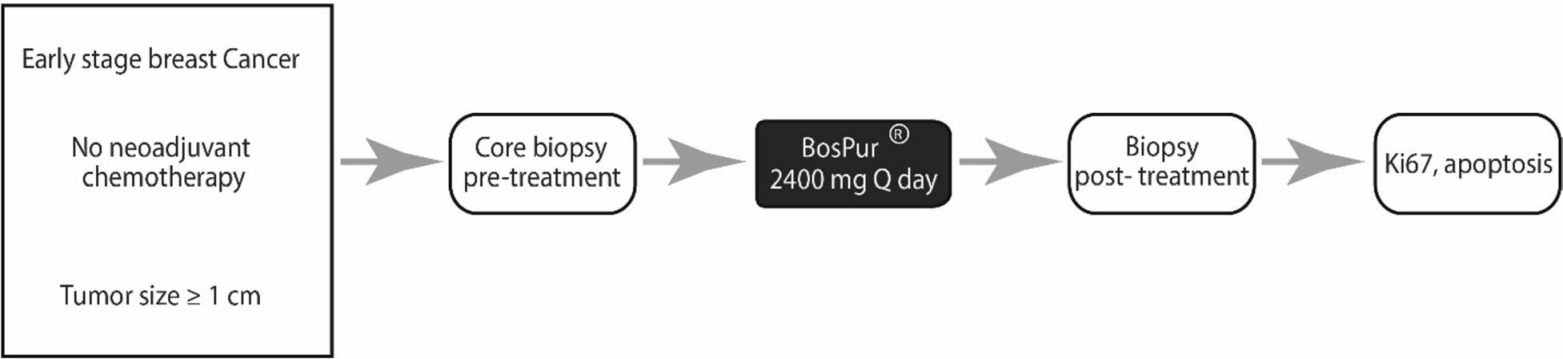
Study design. Twenty-two patients were enrolled on trial, of which 20 received *B. serrata* pre-operatively for 5–56 days until the night before surgery. Eighteen patients had tissue evaluable for IHC. A non-intervention control arm was also included consisting of tissue specimens from matched untreated patients obtained retrospectively

All potential study patients with a core biopsy confirmed invasive breast cancer were evaluated at initial consultation by one of four breast surgeons (AA, DJC, ML, NKD) as per the study design (Fig. 2). The surgeon decided whether the patient was eligible based on tumor size and plan for surgery first. Patients who were screened and deemed potentially eligible had a pathologist (LS) assess for adequate tissue on the diagnostic core biopsy. After informed consent, patients were given BosPure® 400 mg and instructed to take two capsules by mouth three times a day (morning, afternoon, and evening) with food. The drug was taken approximately 6–8 h apart for a total dose of 2400 mg/day. Patients were required to track daily doses of study drugs by maintaining a daily medication diary and to report any side effects. During the first week of drug administration, patients were contacted by a member of the study team who assessed medication compliance and toxicity. If pills were missed, patients were allowed to take them as long as the next dose was due in more than four hours. The skipped dose and the reason for missing were documented in the drug diary. Missed doses were not replaced. The last dose of BosPure® was taken the night before surgery.

### Immunohistochemistry

Formalin fixed, paraffin embedded breast cancer tissue sections were deparaffinized in xylene and hydrated in absolute ethanol, 95% ethanol, and then tap water. Slides were incubated in 3% hydrogen peroxide for ten minutes at room temperature followed by washes in phosphate buffered saline (PBS, Corning, Manassas, VA, cat # 46–013-CM). A citrate buffer antigen retrieval step was performed in a vegetable steamer using the kit Vector Antigen Retrieval Citrate Buffer pH6 (Vector Labs, Newark, CA, cat # H-3300) for 40 min with 10 min to cool. Slides were incubated in blocking serum provided in the Vector Rabbit IMPRESS HRP Kit (Vector Labs, Newark, CA cat # MP-4100) in a humidified slide chamber at room temperature for one hour. The blocking serum was then drained off, and the slides were incubated overnight at 4 °C with the Ki-67 antibody 1:40 dilution (Biocare Medical, Pacheco, CA, cat # PA1-21520). The next day, the slides were rinsed with PBS 1X. The secondary antibody from the Vector Rabbit IMPRESS HRP Kit was added and the slides were incubated for 30 min at room temperature and then rinsed with PBS. A solution of 3,3′-diaminobenzidine (DAB) was prepared and added to the slides as instructed in the Vector DAB kit (Vector Labs, Newark, CA, cat # SK-4100) for five minutes, rinsed in PBS, and counterstained with hematoxylin (Vector Labs, Newark, CA, cat # H-3401) for 30 s. Slides were then washed in distilled water, followed by ammonia alcohol, dehydrated in 95% ethanol, 100% ethanol, xylene, and then cover slipped. The slides were reviewed at 20 × independently for Ki-67 staining by two board-certified pathologists (LS, JF), blinded to control versus treatment group. Both pathologists reviewed all cases. Representative areas of tumor filling the visual field (area of 0.95 mm^2^) were evaluated for Ki-67 positivity. Ki-67 was counted in four fields of representative tumor, when available, and raw counts were provided for statistical evaluation. The areas chosen for counting were representative of the overall sample (global counting, not hot spots). The tumor was not divided into quadrants, rather bias was given to areas where tumor filled the field. For ki67, a direct count of positive tumor cells was used. Positive cells were considered positive if they were moderate staining or better. This technique was applied for both the core and the resection specimens.

TUNEL assay was performed for apoptosis analysis on breast tumor sections following the manual in the Apoptag® Peroxidase In Situ Apoptosis Detection Kit (EMD Millipore, Burlington MA, cat #S7100). All sections were deparaffinized with Histoclear (National Diagnostics, Atlanta, GA, cat# HS-200). The following materials were not supplied with the TUNEL kit and were purchased separately: 30% hydrogen peroxide (JT Baker, Radnor, PA, cat# 5155-01), Proteinase K (Millipore, Burlington MA, cat# 21627), Metal enhanced DAB substrate kit (Thermo Scientific, Rockford, IL, cat# 34065), stable peroxidase substrate buffer 1X (Thermo Scientific, Rockford, IL, cat# 1855910) and 1-butanol (Fisher Scientific, Fair Lawn, NJ, cat# B7908). Images were acquired using the EVOS FLc microscope (Life Technologies, Carlsbad, CA). Tumor apoptosis was quantified as the number of apoptotic nuclei/total tumor nuclei (40×) counting manually four by NKD in four fields of representative tumor, and raw counts were provided for statistical evaluation. This technique was applied for both the core and the resection specimens, and the counting was performed blinded to the control versus treatment group.

### Breast cancer proliferation in vitro

MCF7 cells were cultured in Eagle’s Minimum Essential Medium (Corning #10-009-CV) with 10% fetal bovine serum (FBS) (Biofluid #BT-201-500-D), 10 ng/mL insulin (Sigma #I0516), and Antibiotic Antimycotic solution (Corning 30-004-CI). MDA-MB-231 cells were cultured in Dulbecco’s Modified Eagle’s Medium (Corning #10-013-CV), 10% FBS and Antibiotic Antimycotic solution. For the proliferation study, MCF7 and MDA-MB-231 cells were plated in a 96 well plate at 3000 and 2500 cells/well, respectively. Once the cells settled overnight, they were treated with a concentration curve of BosPure® (in DMSO, 1 μM to 20 μM) or vehicle alone (DMSO 1:250). After 48 h of treatment, the number of healthy cells in each well was assessed using Cyquant Direct Cell Proliferation Assay (Life Technologies Corporation, Eugene, OR, Cat# C35011). One way ANOVA was used to determine statistical significance between the treatment groups. The EC_50_ was determined using an online calculator (https://www.aatbio.com/tools/ec50-calculator).

### Statistical analysis

For Ki-67 analysis, the scores from each assessor for corresponding samples were examined for inter-rater variability using intraclass correlation tests. The change in Ki-67 staining from core specimens to core excision was determined as a percentage and compared between the control and treatment groups using a two-tailed paired t-test. Differences in TUNEL staining between the control and treatment groups were tested for statistical significance using a two-sided t-test. Values are presented as Mean ± standard error of the mean (SEM). Statistical tests were performed using the STATA package (v16.1, College Station, TX). Values of p less than 0.05 were considered to be statistically significant.

## Results

### Study population and characteristics

Twenty-two subjects with invasive breast cancer were enrolled between August 2017 and March 2019. Two patients did not receive the study drug (one subject was deemed ineligible after registration, and one subject withdrew from study), and two patients were excluded after treatment for tissue not sufficient for IHC, for a total of 18 evaluable patients. The demographics and characteristics of the 18 patients that started *B. serrata* and completed the study are shown in Table 2. The median age was 60.1 (range 46–81); 16.7% of patients were African American, 77.8% Caucasian, 5.6% other. The mean duration of drug intake was 11 days (SD 6 days; range: 5–23 days). All patients were pathologic stage I or II. Although the study was not restricted to endocrine receptor tumors, all patients in the study had estrogen receptor (ER) or progesterone receptor (PR) positive breast cancer, and 16 of 18 were Her2neu negative. In the control group, all patients were endocrine receptor positive, Her2neu negative, and pathologic stage I or II.

**Table 2:** Demographics and tumor characteristics of the evaluable patients

Demographics	Number of patients	Percent of patients
Age		
Mean (Range)	60.1 (46–81)	
< 50	3	16
≥ 50	15	84
Race		
Black/African–American	3	16.7
White	14	77.8
Other	1	5.6
Ethnicity		
Non-hispanic	17	94.4
Hispanic	0	0
Unknown	1	5.6
Estrogen or progesterone		
Positive	18	100
Negative	0	0
Her2 neu		
Positive	2	11.1
Negative	16	88.9
Primary tumor stage	N	%
pT1	12	66.7
pT2	6	33.3
Regional lymph node stage	N	%
pN0	18	100
pN1	0	0
Tumor grade		
G1	9	50.0
G2	8	44.4
G3	1	5.6

## Primary outcome measures

### Change in tumor Ki-67 and apoptosis

**Fig. 3:**
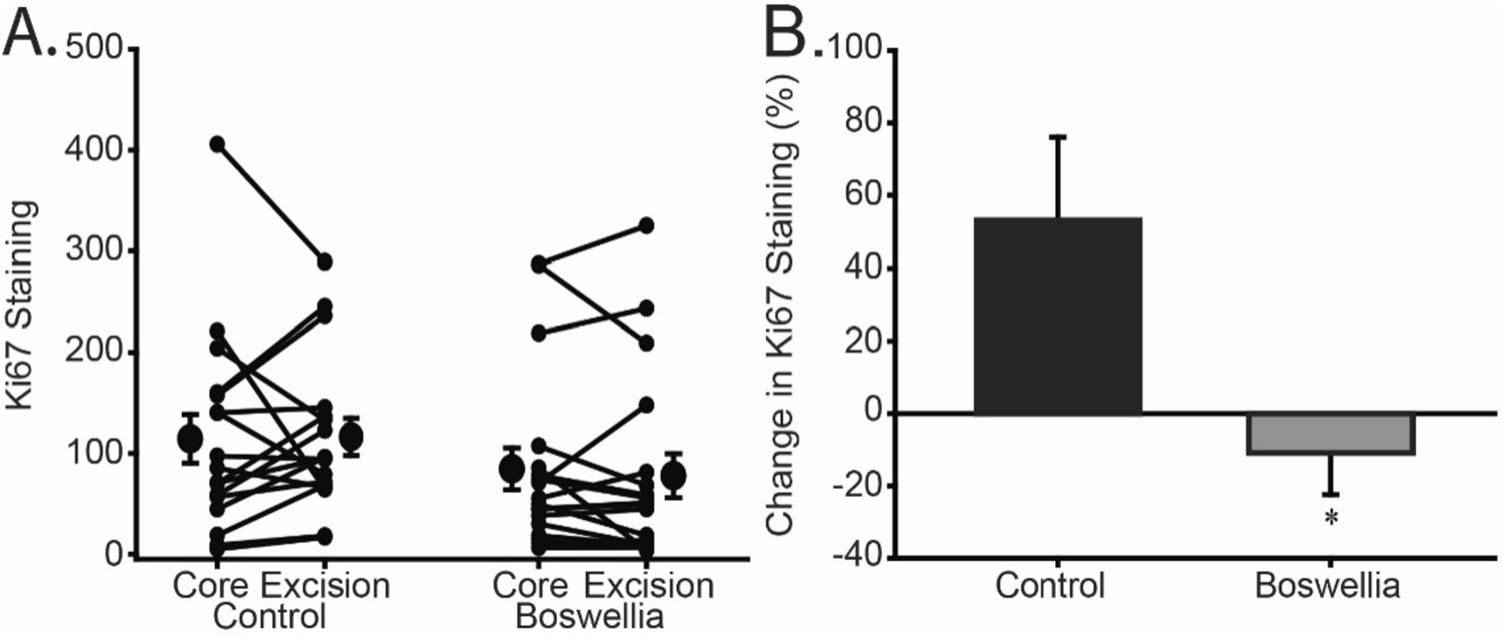
**a** Percent change in proliferation between core biopsy and surgical excision in control patients and patients treated with *B. serrata* as scored by two pathologists. Data points represent the average of the two scores. There was an increase in percent change in proliferation from core biopsy to excisional biopsy in the control group (n = 18) of 54.6 ± 21.4%. In the *B. serrata* treated group, there was a reduction in proliferation between core biopsy and excision (n = 18) of 13.8 ± 11.7%. The difference in Ki-67 staining between core and surgical specimens was statistically significant between the control and Boswellia groups (p = 0.008). **b** Individual matched data points of core biopsy and surgical excision tumor KI67 for control and *B. serrata* groups

Two of twenty patients in both control and treated groups were excluded because of technical difficulty with immunohistochemistry (IHC) in the core biopsy, leaving 18 patients for analyses. Proliferation was determined as a function of Ki-67 immunostaining in histological samples. Scoring of Ki-67 immunostaining was performed independently by two pathologists who were blinded with respect to control versus treated group. The measurements from the two pathologists had an intraclass correlation *r* = 0.63, *p* < 0.001, and there was a significant intraclass correlation between the observers (*r* = 0.68, *p* < 0.001). Changes in proliferation before and after treatment with *B. serrata* were measured as the paired difference between Ki-67 IHC staining in core biopsy and surgical specimens and compared to values obtained from the control set of patients, and presented as the average of the two scores from the pathologists (Fig. 3). The percent change in proliferation from core biopsy to excisional biopsy in the control group (n = 18) was an increase of 54.6 ± 21.4%. In the *B. serrata* group, there was a reduction in proliferation between core biopsy and excision (*n* = 18) of 13.8 ± 11.7%. The difference in Ki-67 staining between core and surgical specimens was statistically significant between the control and *B. serrata* groups (*p* = 0.008). There was no significant interaction between *B. serrata* treatment effect and duration of treatment (core vs. excision; *p* = 0.80). Changes in cellular apoptosis, determined by TUNEL staining/HPF, between the core and surgical specimens were similar in the control and *B. serrata* groups (81.4 ± 57.3 vs. 73.0 ± 66.6%, respectively, *p* = 0.92).

### Adverse events

**Fig. 4:**
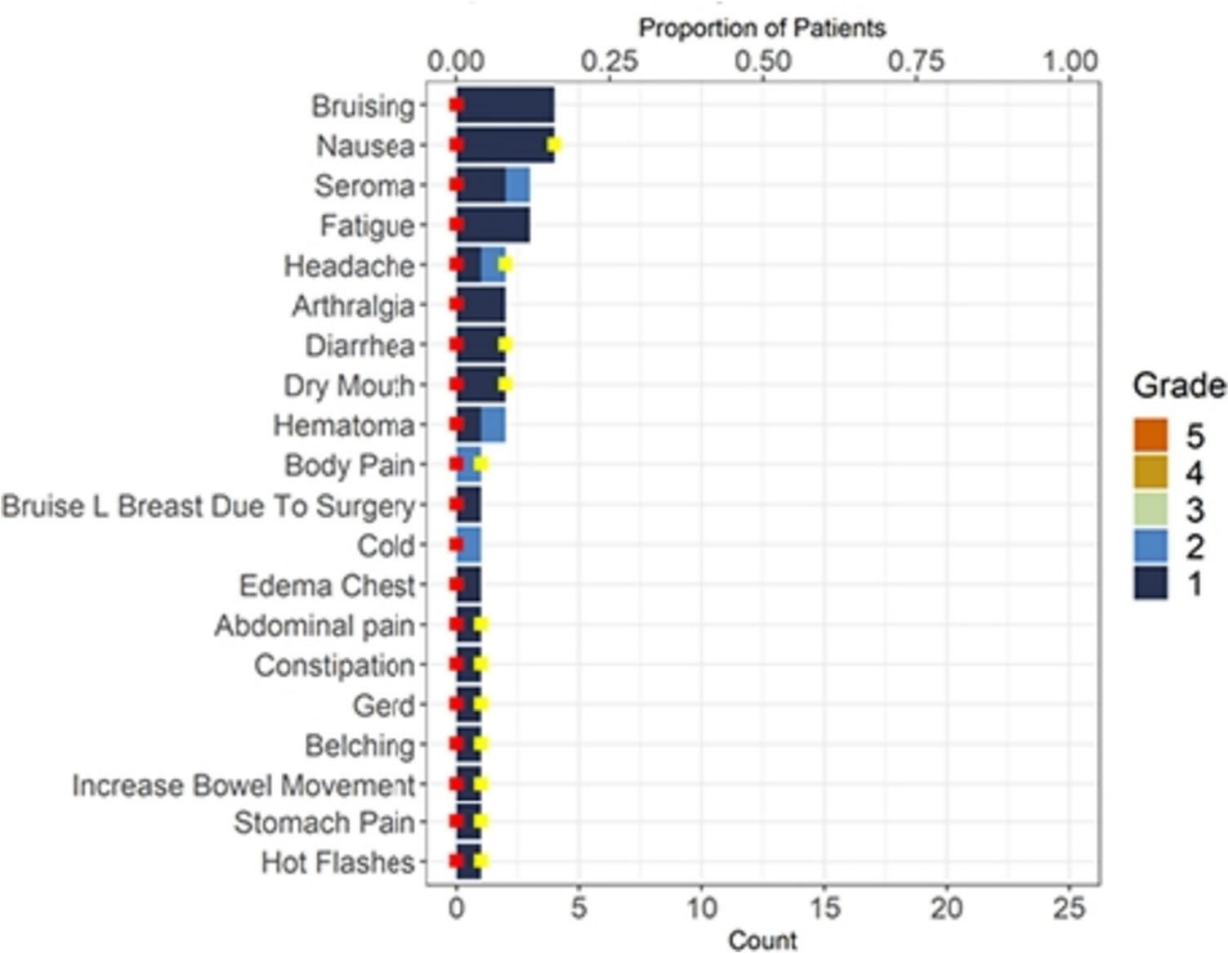
Top 20 adverse events (AE). The red square indicates the serious adverse event count. The yellow square indicates the related/possibly related AE count

There was one Grade 3 adverse event reported on trial (intraoperative hypotension – not related to study drug); however, the majority of toxicities reported were Grade 1. The most prevalent adverse events (AEs) are reported in Fig. 4. There were no serious adverse events reported.

### Changes in tumor proliferation in vitro

**Fig. 5:**
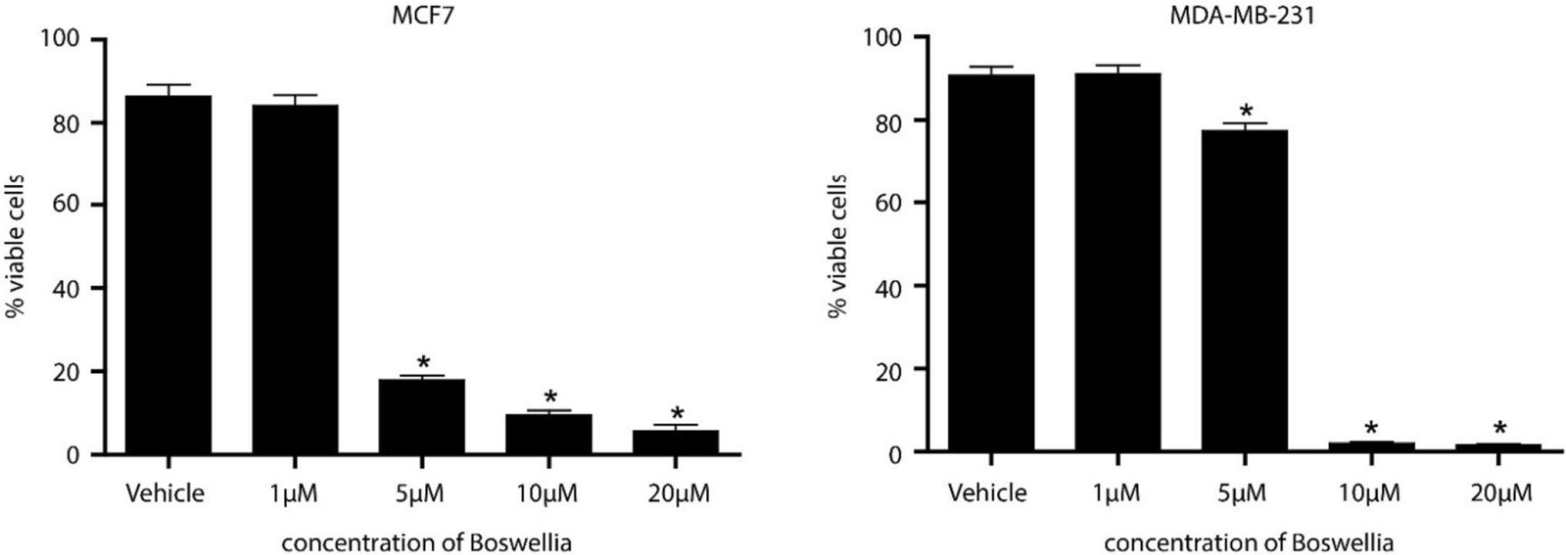
Proliferation studies were conducted to determine the in vitro effect of *B. serrata* (1 μM to 20 μM) on ER + breast cancer cells (MCF7) and triple negative breast cancer cells (MDA-MB-231). Both cells types responded to treatment at concentrations as low as 5 μM (P < 0.0001, n = 12). The EC_50_ of *B. serrata* on MCF7 cells is 2.8 μM and on MDA-MB-232 cells is 5.9Phase μM

Since the clinical trial showed a reduction in tumor proliferation in humans, we wanted to determine if *B. serrata* has a direct effect on proliferation of breast cancer cells in vitro. MCF7 (ER positive) and MDA-MB-231 (triple negative) cells were treated with 1, 5, 10, and 20 μM *B. serrata* resuspended in DMSO. *B. serrata* inhibited both MCF7 and MDA-MB-231 breast cancer cell proliferation (Fig. 5), *n* = 12 per group, demonstrating a direct anti-proliferative effect of *B. serrata* on tumor cells.

## Discussion

Although the clinical use of complementary medicine with plant natural products is growing among patients, there remains very little evidence of efficacy and safety for this class of potential medications in humans. In this Phase Ia window of opportunity study, *B. serrata* significantly decreased tumor cell proliferation in humans with breast cancer, and i*n vitro* in both endocrine ER positive breast cancer cells and triple negative breast cancer cells. *B. serrata* was well tolerated in patients with breast cancer, with no SAEs reported due to study drug. The decrease in tumor proliferation compared to control seen in this study is consistent with several pre-clinical studies that showed an anti-proliferative effect of Boswellia acids on meningioma [14], and leukemia [15]. There have been several preclinical studies have also shown pro-apoptotic effects on cancer cells (breast and colon cancer, melanoma and fibrosarcoma) with Boswellia treatment [16–18]. However, in this clinical trial, there was no difference in tumor apoptosis between pretreatment core breast biopsies compared to excisional breast biopsies after treatment with *B. serrata* in humans. The lack of apoptotic effect seen in vivo in this study may be a function of treatment time or biology of the tumor treated.

The decrease in proliferation seen in the treatment group was in contrast to the increase in proliferation observed between the core biopsy and surgical excision in the control group. This effect has previously been seen in other window of opportunity trials and is explained by a wound healing effect [33]. Morrogh et al. found that control patients had differentially expressed genes between core and excision tumors, including genes involved in cell proliferation. Their IHC analysis confirmed parallel changes in proliferation [33]. In this study, *B. serrata* not only prevented this increase in tumor proliferation, but it decreased the baseline proliferative character of the tumor, which was reduced compared to the control group. Preclinical studies in mouse models have similarly shown a decrease in tumor proliferation with treatment of boswellic acids for different cancers[34].

Although a direct anti-proliferative effect was also seen on both ER positive and triple negative breast cancer cells in vitro, the patients enrolled in this trial were all endocrine receptor positive, and only three patients were Her2neu positive. Although we did not restrict eligibility type, patients who received neoadjuvant chemotherapy were excluded. The majority of patients with triple negative and Her2neu positive breast cancer receive neoadjuvant chemotherapy [35], and neoadjuvant chemotherapy was an exclusion criterion for participation in this trial, which explains why these patients were not represented on this trial. Future studies are needed with a different clinical trial design to evaluate if *B. serrata* reduces breast cancer proliferation in humans with triple negative or Her2neu positive breast cancer.

There have been at least four published clinical trials using *B. serrata* in patients with cancer. Patient with astrocytoma [36], malignant cerebral tumors [37], pediatric progressive or relapsed brain tumors [38], and glioblastoma multiform [39] showed a reduction in cerebral edema. The present study is the first evidence to our knowledge of an in vivo anti-tumor effect of a frankincense extract in breast cancer in humans.

## Conclusion

Overall, this trial demonstrated that oral administration of *B. serrata* at a dose of 2400 mg daily for 5–56 days has a good safety profile and reduced breast tumor cell proliferation in humans in a Phase Ia window of opportunity trial and was well tolerated. This correlates with the reduction in proliferation of breast cancer cell lines treated with *B.serrata *in vitro*.* The purpose of window of opportunity trials is to provide a relatively inexpensive and fast way to determine whether a novel therapeutic has biologic activity in humans. Biologic active of *B. serrata* in humans was demonstrated in this study. Further research is now needed to determine the most active metabolites of *B. serrata* to increase potency, which will be the goal of future studies, and to evaluate whether *B. serrata* reduces local recurrence or improves survival are warranted.

## Data Availability

The datasets used and/or analyzed during the current study are available from the corresponding author on reasonable request.

## Abbreviations

Aes: Adverse events
*B. serrata*: *Boswellia serrata*
DAB: 3,3′-Diaminobenzidine
ER: Estrogen receptor
FBS: Fetal bovine serum
IHC: Immunohistochemistry
AKBA: 3-O-Acetyl-11-keto-β-boswellic acid
PR: Progesterone receptor
SEM: Standard error of the mean
VEGFR2: Vascular endothelial growth receptor 2
